# Chronic Viral Hepatitis and Metabolic Syndrome/Cardiovascular Risk

**DOI:** 10.1155/2018/7369314

**Published:** 2018-11-04

**Authors:** Peter Jarcuska, Ahmed Abdel-Razik, Robert Flisiak, Ram B. Singh

**Affiliations:** ^1^1st Department of Internal Medicine, PJ Safarik University, Faculty of Medicine and L Pasteur University Hospital, Kosice, Slovakia; ^2^Tropical Medicine Department, Faculty of Medicine, Mansoura University, Mansoura, Egypt; ^3^Department of Infectious Diseases and Hepatology, Medical University of Białystok, Białystok, Poland; ^4^Halberg Hospital and Research Institute, Moradabad, India

Chronic viral hepatitis B and C can lead to liver cirrhosis. Hepatocellular carcinoma (HCC) is another complication, however, and, particularly interestingly, it can occur not only in patients with advanced liver fibrosis or cirrhosis, but also in patients with chronic hepatitis B infection and high viremia. Both decompensated liver cirrhosis and HCC lead to death in patients with chronic viral hepatitis B or C. There are several factors, including hepatic comorbidities such as nonalcoholic fatty liver disease (NAFLD) or alcoholic liver disease, which accelerate liver fibrosis in patients with chronic viral hepatitis [1].

Metabolic syndrome (MetS) is associated with insulin resistance. MetS is present more frequently in obese patients or in patients with type 2 diabetes mellitus (T2DM). Patients with MetS have significantly higher both cardiovascular morbidity and mortality compared to those without MetS. MetS is present in approximately 25% of population in advanced countries, while its prevalence demonstrates increasing trend with higher age [2]. MetS prevalence depends on many factors, including viral infection.

Cardiovascular risk is not higher in patients with chronic viral hepatitis B, nor is the risk of hyperlipidemia, T2DM, or MetS. On the other hand, MetS or NAFLD comorbidities accelerate liver fibrosis and increase HCC incidence among patients with viral hepatitis B [3].

Patients with chronic viral hepatitis C are being diagnosed more frequently with higher insulin resistance, prediabetes, and T2DM. T2DM occurrence is rising with stage of liver fibrosis and is very high in patients who failed to reach sustained viral response (SVR) after antiviral therapy was completed [4, 5]. Liver steatosis is present more frequently in patients with chronic hepatitis C. Viral steatosis is specific condition characterized by higher hepatocellular fat accumulation without higher insulin resistance in patients infected mainly with genotype 3 [5]. MetS prevalence is not higher in population of patients infected with hepatitis C virus, which has both higher T2DM prevalence and insulin resistance, compared to population of never infected patients. The reason is most likely that chronic hepatitis C patients do not have atherogenic hyperlipidemia [6]. Increased peripheral and hepatic insulin resistance, chronic inflammation, chronic endothelial injury, and direct viral effect on arterial wall most likely also lead to accelerated atherogenesis. Patients with chronic hepatitis C have higher cardiovascular risk compared to those never infected, together with increased prevalence of coronary artery disease, unstable angina pectoris, myocardial infarction, and stroke [7]. Interestingly, SVR achievement after interferon based treatment in patients with chronic hepatitis C reduced T2DM incidence in the future. Nevertheless, risk of T2DM development after SVR achievement is higher in patients with BMI>25 [4]. Nowadays, there is highly effective treatment of chronic hepatitis C. Nearly all patients achieve SVR by the treatment with direct acting antivirals (DAA); moreover, DAA therapy has very low occurrence of serious adverse events and is thus considered safe [8]. DAA therapy leads to both significant decrease in fasting glucose (FG) levels and significant decrease in glycated hemoglobin levels (HbA1C) in diabetic patients with chronic hepatitis C and is thus requiring reduction of antidiabetic therapy in certain part of patients [9]. Successful DAA therapy will most likely lead to drop in both T2DM prevalence and cardiovascular risk, making it essential to remove all barriers limiting easy diagnostics and therapy. Patients with chronic viral hepatitis could also benefit from statin therapy for its antifibrotic and antineoplastic effect [10].

S. Drazilova et al. described in the review article pathophysiological mechanisms of both increased peripheral and hepatic insulin resistance. Authors also evaluated predictive factors of T2DM. Diabetes mellitus is present more frequently in patients with chronic hepatitis C and cirrhosis, while it also predicts liver decompensation. DAA treatment led to decrease in FG levels and/or HbA1C levels in nearly all studies, limitation of most of the studies was retrospective design. Approximately 3-40% of diabetic patients with chronic hepatitis C required reduction of antidiabetic therapy during DAA therapy. Patients with chronic hepatitis C rarely have elevated total cholesterol (TC), LDL cholesterol (LDL-C), and triglycerides (TG) levels. However, during DAA therapy, one can await alteration of lipoprotein profile. Studies observed elevation in both TC and LDL-C levels; on the other hand, most of the studies observed both drop in TG and elevation in HDL-C levels during DAA therapy, although, further research into alteration of lipoprotein profile during DAA therapy is still required.

Retrospective analysis from Slovakia (S. Drazilova et al.) evaluated glucose metabolism changes in patients with chronic hepatitis C treated with DAA. Altogether, 370 patients were observed, 45,9% in F4 by Metavir. Risk of T2DM development increases with liver fibrosis stage. T2DM was found in 14.4% patients with F0-F2 fibrosis, 21.3% patients with F3, and 31.8% with F4 fibrosis (p=0.004). FG levels, impaired fasting glucose (IFG) or T2DM prevalence were not significantly different between patients in Child-Pugh A and Child-Pugh B/C stage. Correlations between FG and APRI score were observed (R^2^ = 0.018, p=0.026). Treatment experienced patients had significantly higher FG (p=0.006) and significantly higher prevalence of IFG or T2DM (p=0.005) when compared to treatment naive patients. Age, BMI, and F4 stage of fibrosis by Metavir predicted in univariate analysis T2DM prevalence. DAA treatment led to drop in FG in all patients (p=0.002), patients with IFG (p<0.0001), and T2DM (p<0.0001), although not in patients without IFG or T2DM (p=0.192). Significant drop in FG was observed in all experienced patients (p<0.0001), experienced cirrhotics (p<0.0001), although not in treatment naive patients. Drop in FG was observed in all cirrhotic patients (p=0.009), however not in patients with Child-Pugh score B/C (p=0.568). Significant change in FG levels during and after DAA therapy was not observed in patients with liver fibrosis F0-F3 by Metavir. Baseline FG was only predictor of significant decrease of glycemia (>5%) after DAA treatment (p<0.0001).

S. Mustapic et al. correlated ultrasound grade of liver steatosis with the risk of MetS. Authors evaluated 159 patients, 34% of them were obese. Patients with higher ultrasound grade of steatosis had significantly higher BMI, increased prevalence of obesity, impaired glucose metabolism, atherogenic dyslipidemia, raised blood pressure and significantly more frequently met the modified criteria of MetS (P<0.05 for all analyses). Ultrasound grade of liver steatosis was significantly independently associated with the presence of MetS (p=0.007 for moderate-to-severe liver steatosis). Authors did not find significant difference in FIB4 value in different ultrasound grades of liver steatosis (p=0.251).

M. Flisiak-Jackiewicz et al. studied predictive role of interleukin-18 (IL-18) in liver steatosis in obese children. IL-18 correlates with ALT (p=0.036), AST (p=0.032), GGT (p=0.016), TG (p=0.027), hs-CRP (p=0.014), ultrasound grade of liver steatosis (p=0.044), and waist circumference (p=0.009). Level of IL-18 was higher in obese children with advanced liver steatosis found on ultrasound examination compared to children without steatosis (p=0.027). The concentration of IL-18 was significantly higher in obese children with steatosis found on magnetic resonance proton spectroscopy (^1^HMRS) compared to children without fatty liver (p=0.014). IL-18 will most likely be used as NAFLD predictor in obese children in the future.

T. Stroffolini et al. described characteristics and changes over time of alcohol-related chronic liver diseases in Italy. Authors enrolled 12 256 subjects from two national surveys made in 2001 and 2014. 2 717 (22.2%) cases had a risky alcohol intake; 48.3% of them were anti HCV positive. Sex ratio (male/female) decreased from 3.8 in 2001 to 1.3 in 2014 and women were significantly older than men (58.9 versus 53.1 years; p<0.01). The proportion of subjects with liver cirrhosis increased over time in both sexes, and decompensation of liver cirrhosis (Child B or C) was found in approximately half of cases. Risky alcohol drinking plays important role in chronic liver diseases in Italy.

Studies mentioned above highlight that the association between chronic hepatitis C and T2DM is a very serious medical issue, which is present mainly in advanced chronic hepatitis C, in patients with liver cirrhosis or in experienced patients. Furthermore, studies also highlight that DAA therapy leads to drop in FG. Liver steatosis and risky alcohol intake can eventually deteriorate clinical course of chronic viral hepatitis, making it necessary to diagnose all simultaneous hepatic comorbidities as early as possible, eventually to look for surrogate markers (such as IL-18) for liver fat accumulation diagnostics in patients at risk.
